# Sexual transmission of Zika virus and other flaviviruses: A living systematic review

**DOI:** 10.1371/journal.pmed.1002611

**Published:** 2018-07-24

**Authors:** Michel Jacques Counotte, Caron Rahn Kim, Jingying Wang, Kyle Bernstein, Carolyn D. Deal, Nathalie Jeanne Nicole Broutet, Nicola Low

**Affiliations:** 1 Institute of Social and Preventive Medicine, University of Bern, Bern, Switzerland; 2 Department of Reproductive Health and Research, World Health Organization, Geneva, Switzerland; 3 Division of Sexually Transmitted Disease Prevention, Centers for Disease Control and Prevention, Atlanta, Georgia, United States of America; 4 National Institute of Allergy and Infectious Diseases, Bethesda, Maryland, United States of America; Mahidol-Oxford Tropical Medicine Research Unit, THAILAND

## Abstract

**Background:**

Health authorities in the United States and Europe reported an increasing number of travel-associated episodes of sexual transmission of Zika virus (ZIKV) following the 2015–2017 ZIKV outbreak. This, and other scientific evidence, suggests that ZIKV is sexually transmissible in addition to having its primary mosquito-borne route. The objective of this systematic review and evidence synthesis was to clarify the epidemiology of sexually transmitted ZIKV.

**Methods and findings:**

We performed a living (i.e., continually updated) systematic review of evidence published up to 15 April 2018 about sexual transmission of ZIKV and other arthropod-borne flaviviruses in humans and other animals. We defined 7 key elements of ZIKV sexual transmission for which we extracted data: (1) rectal and vaginal susceptibility to infection, (2) incubation period following sexual transmission, (3) serial interval between the onset of symptoms in a primary and secondary infected individuals, (4) duration of infectiousness, (5) reproduction number, (6) probability of transmission per sex act, and (7) transmission rate. We identified 1,227 unique publications and included 128, of which 77 presented data on humans and 51 presented data on animals. Laboratory experiments confirm that rectal and vaginal mucosae are susceptible to infection with ZIKV and that the testis serves as a reservoir for the virus in animal models. Sexual transmission was reported in 36 human couples: 34/36 of these involved male-to-female sexual transmission. The median serial symptom onset interval in 15 couples was 12 days (interquartile range: 10–14.5); the maximum was 44 days. We found evidence from 2 prospective cohorts that ZIKV RNA is present in human semen with a median duration of 34 days (95% CI: 28–41 days) and 35 days (no CI given) (low certainty of evidence, according to GRADE). Aggregated data about detection of ZIKV RNA from 37 case reports and case series indicate a median duration of detection of ZIKV of 40 days (95% CI: 30–49 days) and maximum duration of 370 days in semen. In human vaginal fluid, median duration was 14 days (95% CI: 7–20 days) and maximum duration was 37 days (very low certainty). Infectious virus in human semen was detected for a median duration of 12 days (95% CI: 1–21 days) and maximum of 69 days. Modelling studies indicate that the reproduction number is below 1 (very low certainty). Evidence was lacking to estimate the incubation period or the transmission rate. Evidence on sexual transmission of other flaviviruses was scarce. The certainty of the evidence is limited because of uncontrolled residual bias.

**Conclusions:**

The living systematic review and sexual transmission framework allowed us to assess evidence about the risk of sexual transmission of ZIKV. ZIKV is more likely transmitted from men to women than from women to men. For other flaviviruses, evidence of sexual transmissibility is still absent. Taking into account all available data about the duration of detection of ZIKV in culture and from the serial interval, our findings suggest that the infectious period for sexual transmission of ZIKV is shorter than estimates from the earliest post-outbreak studies, which were based on reverse transcription PCR alone.

## Abstract 2

In a systematic review of human and animal studies, Nicola Low and colleagues use a novel framework to characterize the risks of human sexual transmission of ZIKV and other arthropod-borne flaviviruses.

## Author summary

**Why was this study done?:**

- Sexual transmission of Zika virus (ZIKV) is now documented, but the risks of transmission are not well understood.
- It is not known whether other flaviviruses can be transmitted through sexual intercourse.

**What did the researchers do and find?:**

- We developed a sexual transmission framework for ZIKV infection that identified 7 key elements related to ZIKV sexual transmission, and we conducted a living systematic review through 15 April 2018 of available evidence about each element.
- We found that, where documented, sexual transmission of ZIKV is much more common from men to women than from women to men. For sexual transmission of ZIKV, the median serial interval—the time between onset of symptoms in 2 sexual partners—is 12 days.
- The median duration of ZIKV RNA persistence in semen is longer (34 days) than in the female genital tract (12 days). ZIKV can be detected for longer periods using reverse transcription polymerase chain reaction compared to viral culture.
- We found no evidence of sexual transmission for any other arthropod-borne flaviviruses.

**What do these findings mean?:**

- Studies about the duration of detection of ZIKV in bodily fluids and the serial interval suggest that the period ZIKV can be transmitted through sexual contact might be shorter than was estimated from the earliest studies in 2016.

## Introduction

Zika virus (ZIKV) can be transmitted between humans through sexual contact, although it is most commonly transmitted by infected *Aedes* spp. mosquitoes [1,2]. Sexual transmission of ZIKV has important implications for public health, for people living in endemic regions, and for sexual partners of travellers returning to non-endemic regions from endemic regions because ZIKV infection during pregnancy can cause congenital infection of the foetus and because ZIKV infection can trigger the immune-mediated neurological condition Guillain-Barré syndrome [3,4]. ZIKV is an RNA flavivirus. Flaviviruses are a genus of viruses from the Flaviviridae family, of which the majority are transmitted to vertebrates by infected mosquito or tick vectors [5].

Scientists working in Senegal in 2008 were the first to report presumed sexual transmission of ZIKV in a case report that documented their own symptoms and serological findings [6]. One scientist developed symptoms after returning to the US, and his wife, who had not travelled outside the US, became unwell 4 days later. The large ZIKV outbreak (2015–2017) in the Americas resulted in additional reports of travel-associated ZIKV sexual transmission in the US and Europe, which Moreira and colleagues synthesised descriptively in a systematic review of the literature up to December 2016 [7]. In vivo and in vitro experimental studies have provided evidence of the biological plausibility of this route of infection [8].

While possible sexual transmission has been established, there are many unanswered questions about the transmissibility of ZIKV through sexual intercourse. For mosquito-borne ZIKV infection, the incubation period and duration of viral shedding in serum have been estimated, allowing implications for blood donation to be assessed [9]. Additional information about parameters related to person-to-person transmission of ZIKV has not been systematically collated or quantified, although several narrative reviews have been published [10,11]. Evidence about sexual transmission of other arthropod-borne flaviviruses in humans, including West Nile virus (WNV), yellow fever virus (YFV), Japanese encephalitis virus (JEV), and dengue virus (DENV) [12], has not been synthesised, but WNV and YFV have been detected in human semen [13,14]. The primary objective of this review was to systematically review evidence about defined aspects of the sexual transmission of ZIKV. Secondary objectives were to systematically review evidence about the sexual transmissibility of other arthropod-borne flaviviruses and to establish these reviews using a living systematic review approach [15].

## Methods

### Sexual transmission framework

**Fig 1:**
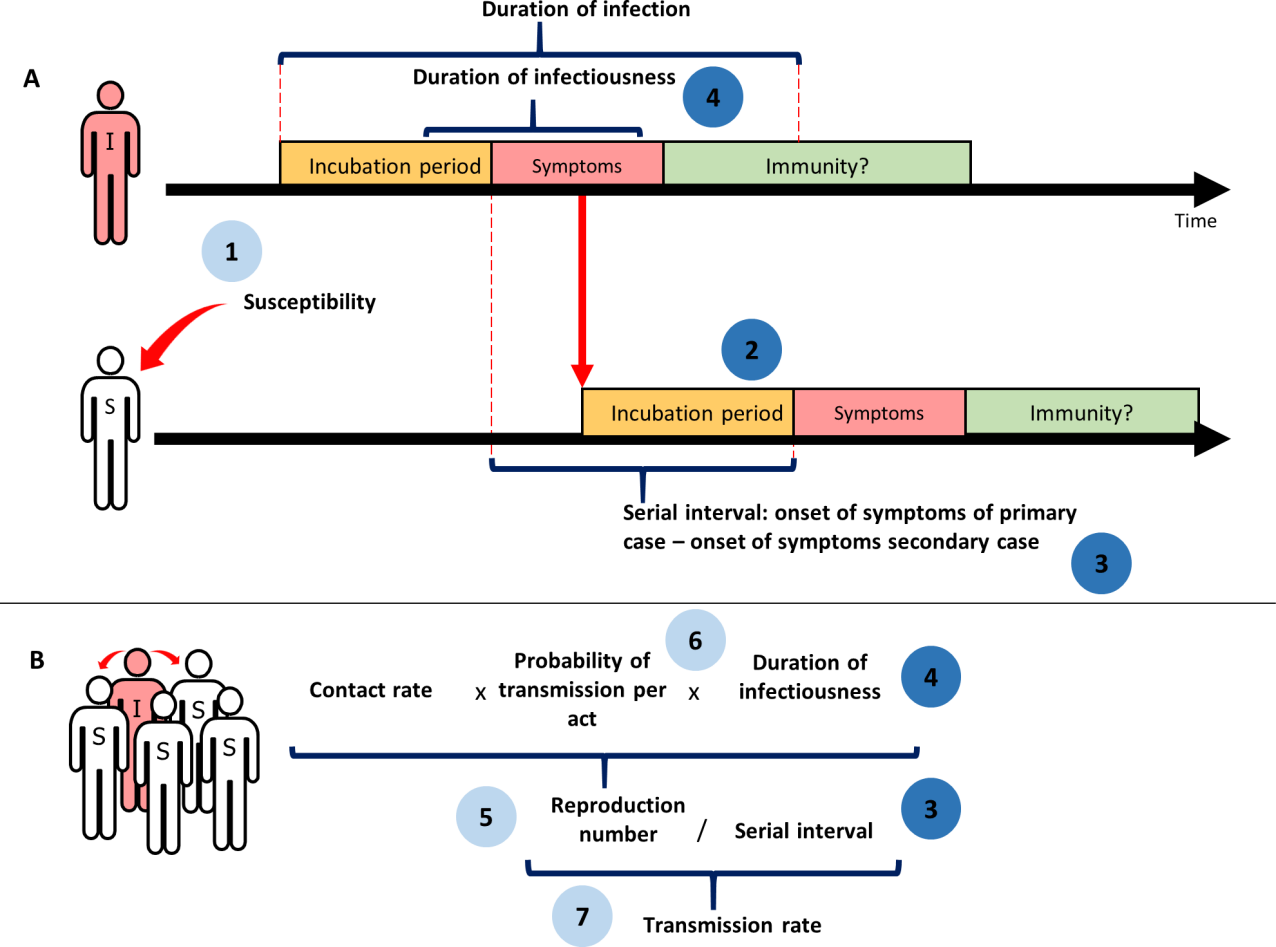
A schematic representation of the sexual transmission of Zika virus and 7 key elements. Numbered circles show the 7 key elements. Dark blue circles are elements for which evidence is based on empirical research. Light blue circles denote elements derived from mathematical modelling studies and in vivo studies. (A) Transmission between 2 individuals. The horizontal arrows show the time course of the disease for the primary infected individual (I), who is infected, and the secondary individual (S), who starts as susceptible (element 1). The vertical red arrow represents a Zika virus transmission event, after which there is an incubation period (element 2) before symptoms develop. Element 3 is the serial interval, i.e., the period between the start of symptoms in the primary and the secondary individual. Element 4 is the duration of infectiousness. After the infection, individuals can become immune. (B) Relation between different elements at population level. The reproduction number (element 5) is the result of the contact rate, the probability of transmission per act (element 6), and the duration of infectiousness (4). The transmission rate (element 7) can be estimated using the reproduction number (5) and the serial interval (3). Adapted with permission from World Health Organization [16].

In March 2017, we developed a sexual transmission framework for ZIKV [16,17], based on standard concepts about person-to-person transmission of infection [18]. The framework includes key elements in the course of an infection in an individual and transmission to a sexual partner, some of which can be measured and others that can only be determined indirectly or through modelling. Fig 1 shows these elements and the relationships between them: (1) susceptibility to infection, (2) incubation period after sexual transmission, (3) serial interval, (4) duration of infectiousness, (5) reproduction number, (6) probability of transmission per sex act, and (7) transmission rate. The framework does not include transmission from and to mosquitoes, which would be needed to estimate the proportion of all ZIKV infections due to sexual transmission. The sexual transmission framework defined the outcomes and informed the structure of the review.

### Living systematic review

We performed this review as a living systematic review [15] because research into many aspects of ZIKV is a new and fast-moving field. Several studies are ongoing [19] and have published interim results [20], and updated results could affect public health decisions. The protocol for this review was registered on 19 May 2017 in the database PROSPERO (CRD42017060338) [21]. We summarise the details that make the review a living systematic review in S1 Text. Future updates will be reported quarterly online (http://zika.ispm.unibe.ch/stf/) and in the online comments section of this publication. Reporting is in accordance with the Preferred Reporting Items for Systematic Reviews and Meta-Analyses (S1 PRISMA Checklist).

### Search strategy

The search includes the electronic databases PubMed, Embase, bioRxiv, arXiv, PeerJ, and LILACS and online repositories from the US Centers for Disease Control and Prevention, the European Centre for Disease Prevention and Control, the Pan American Health Organization, and the World Health Organization from the earliest date of each database and without language restrictions. The searches include Medical Subject Headings (MeSH) terms and keywords for ZIKV and flaviviruses together with terms and keywords for viral persistence and sexual transmission (S1 Text). An automated search is run every day, with results deduplicated and imported into REDCap (Research Electronic Data Capture). We checked reference lists of included studies to identify additional relevant studies. For this report, we identified studies published before and up to 15 April 2018.

### Eligibility criteria

We included observational studies, in vitro and in vivo studies, and mathematical modelling studies that directly addressed any of the elements of the sexual transmission framework in either humans or animals for ZIKV or another arthropod-borne flavivirus. We included observational studies that reported 1 or more cases of sexual transmission, 1 or more measurements of presence of virus in bodily fluids, or both. As bodily fluids we included semen, cervical and vaginal secretions, and saliva; diagnostic methods included reverse transcription polymerase chain reaction (RT-PCR) and viral culture. We did not include reviews, editorials, or commentaries that did not report original data. Table 1 provides an overview of the eligibility criteria for each outcome. Primary outcomes can be directly estimated from observational studies, and secondary outcomes are calculated or inferred from indirect evidence.

**Table 1:** Eligibility criteria for each outcome.

Outcomes	Eligible study designs	Detailed eligibility criteria
**Primary outcomes**		
Element 2. Incubation period following sexual transmission	Observational epidemiological studies in humans (case reports, case series, cohort studies, case–control studies, surveillance/outbreak reports)	Observational studies that report incubation period due to sexual transmission
Element 3. Serial interval	Observational epidemiological studies in humans	Observational studies that describe sexual transmission in humans where serial interval (time between onset of symptoms between sexual partners) is reported
Element 4. Duration of infectiousness	Observational epidemiological studies in humans	Observational studies that report duration of detection of virus in semen, cervical and vaginal secretions, and saliva; diagnostic methods included reverse transcription PCR and viral culture
**Secondary outcomes**		
Element 1. Susceptibility	Basic research studies (in vivo/in vitro studies)	In vivo/in vitro studies that report on the presence of virus in the female genital tract, the male genital tract, or saliva, or on sexual transmission of virus
Element 5. Reproduction number due to sexual transmission	Mathematical modelling studies	Modelling studies that report on the elements of interest
Element 6. Probability of transmission per sex act	Mathematical modelling studies	Modelling studies that report on the elements of interest
Element 7. Transmission rate	Mathematical modelling studies	Modelling studies that report on the elements of interest

### Study selection and data extraction

One reviewer screened titles and abstracts of retrieved papers. If retained in the first step, the same reviewer screened the full text of the paper. One reviewer extracted data into piloted extraction forms in REDCap [22]. A second reviewer verified exclusion decisions and data entry.

### Synthesis of the evidence

We provide descriptive summaries of findings about the elements of the ZIKV sexual transmission framework for basic research studies (element 1), observational epidemiological studies (elements 2–4), and mathematical modelling studies (elements 5–7). In addition, we used data from included studies to calculate estimates for the serial interval (i.e., the period between the start of symptoms in the primary and the secondary individual) and the duration of the detection of ZIKV. We report the median serial interval and its interquartile range. To estimate the duration of detection of ZIKV positivity, we conducted interval-censored survival analysis and fitted Weibull distributions using the “straweib” package [23,24] in R (version 3.4.1), based on previous studies [20,23,24]. We assumed that all infected patients were RT-PCR or viral culture positive at symptom onset. We report median estimated durations and corresponding 95% confidence intervals. Additional information about the methods is provided in S2 Text. For other flaviviruses, we summarise findings from all study types descriptively.

### Certainty assessment of the evidence

We assessed the methodology of included studies using specific checklists for each study type. For observational studies, we used the National Institutes of Health (NIH) Quality Assessment Tool for Case Series Studies [25] and UK National Institute for Health and Care Excellence (NICE) checklists for case–control studies and cohort studies [26]. For in vivo studies we used the SYstematic Review Centre for Laboratory animal Experimentation (SYRCLE) risk of bias tool for animal studies [27], and for mathematical modelling studies, the International Society for Pharmacoeconomics and Outcomes Research (ISPOR) Questionnaire to Assess Relevance and Credibility of Modelling Studies [28]. We performed the assessment by a consensus-driven approach among multiple reviewers. We appraised the certainty of the key elements according to the Grading of Recommendations Assessment, Development and Evaluation (GRADE) tool [29–31] (S3 Table). In accordance with GRADE, assessments of the overall certainty of evidence from observational studies started at low certainty. We downgraded the level of certainty for small sample size and evidence from case reports or case series. We assessed outcomes of mathematical modelling studies as high, medium, low, or very low certainty.

## Results

**Fig 2:**
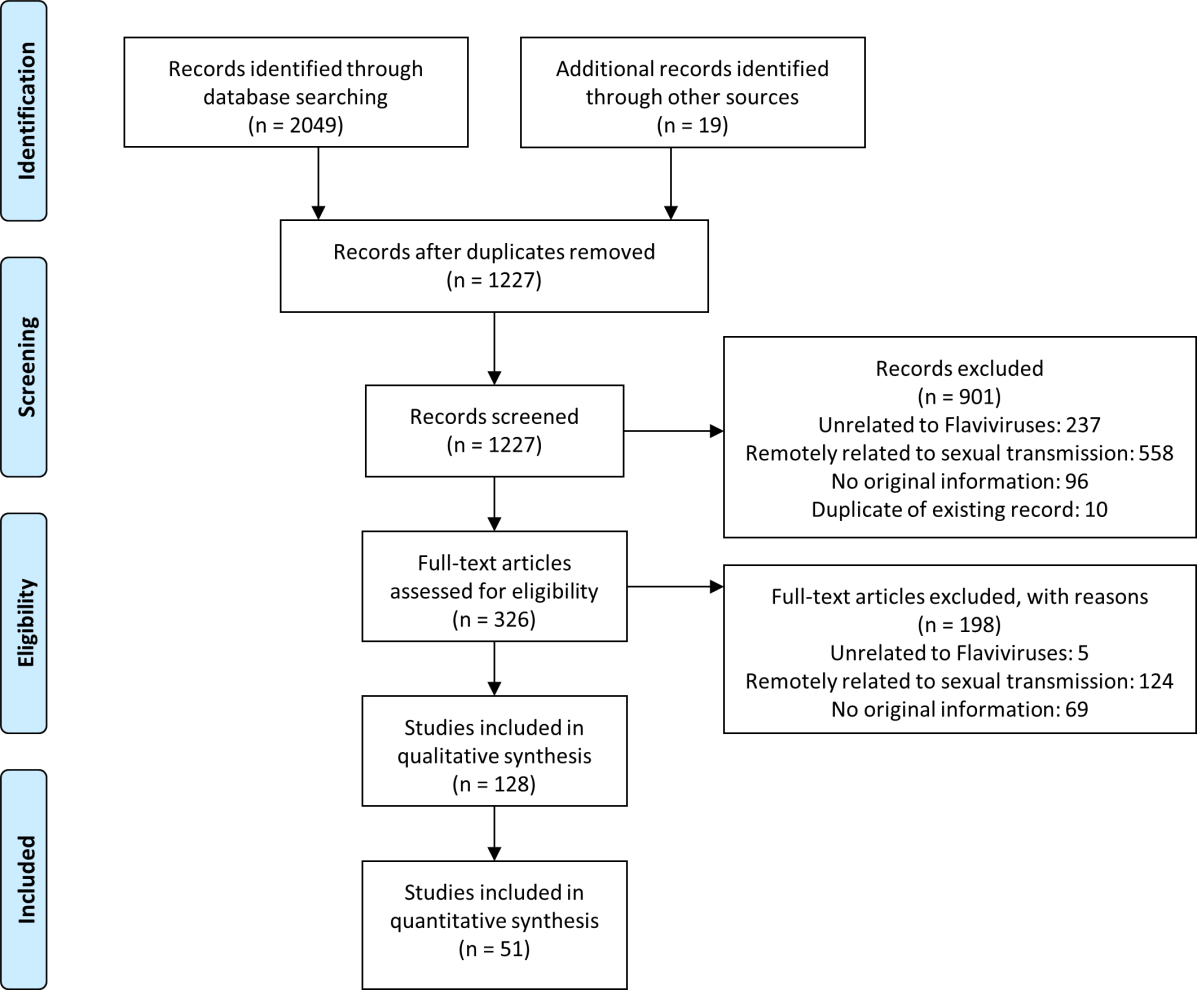
Flow diagram of reviewed studies. Numbers of studies screened, assessed for eligibility, and included in the review, with reasons for exclusions at each stage.

We identified 1,227 unique citations and excluded 901 by title and abstract screening (Fig 2). Of the remaining 326 potentially eligible citations with relevant abstracts, 128 publications were eligible for inclusion. Table 2 summarises characteristics of the included studies.

**Table 2:** Overview of study designs of included studies.

Category	Publications on Zika virus	Publications on other flaviviruses
**Epidemiological studies**		
Case reports	44	7
Case series	18	1
Cohort studies	4	—
Case–control studies	—	—
Outbreak or surveillance reports	1	—
**Mathematical modelling studies**	2	—
**Basic research studies**		
In vivo studies	35	7
In vitro studies	6	1
Review studies	—	2
**Total publications**	110	18
Reporting on sexual transmission between 2 partners	24^a^	1
**Publications used for quantitative analysis**	51	—
Reporting serial interval	12^a^	—
Reporting at least 1 measurement in bodily fluids of interest using reverse transcription PCR or viral culture	48^a^	—

^a^Overlap in publications; one publication can report on multiple outcomes (e.g., reporting on serial interval and/or persistence and/or sexual transmission).

### Basic research studies

We included 41 in vivo and in vitro studies of ZIKV [32–69] (Table 2). Of these 41 studies, 6 were in vitro studies and 35 were studies in in vivo animal models: 12 in nonhuman primates (NHPs) such as cynomolgus macaques (*Macaca fascicularis*), rhesus macaques (*M*. *mulatta*), and common marmosets (*Callithrix jacchus*) and 23 in mice. In 1 study, both guinea pigs and NHPs were used [64]. These studies provide insight into the underlying biological mechanisms of susceptibility to ZIKV infection through sexual transmission and substantiate the biological plausibility of this transmission route.

### Susceptibility (element 1)

In mouse and NHP models, the vaginal and rectal mucosae were shown to be susceptible to infection with ZIKV [36,40,47,49,51,59]. When ZIKV-infected male mice were mated with uninfected female mice, the female mice became infected [47,53,69]. Female-to-male transmission of ZIKV in mice was unsuccessful [47]. In rhesus macaques, systemic infection through oropharyngeal mucosal inoculation with ZIKV was only successful after inoculation with a very high dose of virus, suggesting a very low risk of oral mucosal transmission [54]. Four rhesus macaques became viraemic after intranasal or intragastric inoculation with ZIKV [64]. In guinea pigs, direct transmission between animals infected subcutaneously with ZIKV and co-housed uninfected animals was seen [64].

Human prostate cells, testicular cells, and mature spermatozoa are susceptible in vitro to ZIKV infection [35,60–62]. Human Sertoli cells can support high levels of ZIKV replication and persistence [70]. In multiple mouse models, using different strains of ZIKV, the testes seem to be a preferred site for viral replication, able to sustain high viral loads for a longer duration than other organs [32,34,35,43,47,49,51,58,63]. In some of these models, ZIKV caused inflammation of the testes [35,39,41,47,49], reduced testicular size, and decreased levels of testosterone [49,56,68,71]. The testes of experimentally infected NHPs harboured high levels of ZIKV [37,38]. High titres of ZIKV RNA were detectable in semen until day 28 in rhesus and cynomolgus macaques [38]. However, 1 group of rhesus macaques had only low levels of viral RNA in the testes and no detectable virus in the prostate or epididymis [44]. In common marmosets, ZIKV RNA in semen was sporadically detected [65].

The female genital tract of macaques was able to sustain ZIKV replication for shorter durations and with lower viral loads than the male genital tract [33,38,46]. Although cervical and endometrial cells were susceptible in vitro [35], virus was not detected in the female genital tract in mice or NHPs for longer than 7 days after infection [33,46]; in 1 study, the ovary sustained higher titres up to 14 days post-infection [35]. Intravaginal infection of mice led to systemic infection [36,40,42,47,48,50,67,68,69] and to adverse congenital outcomes [47]. In pregnant female mice, sexual transmission led to more ZIKV dissemination to the female reproductive tract, compared with subcutaneous or intravaginal inoculation [69]. In NHPs the incubation time following infection was longer for intravaginal infection compared with subcutaneous infection [55].

In mice, viral titres were lower in the salivary glands than the testes and ovaries [35]. In NHPs, viral RNA was detected in saliva up to 28–42 days [33,37,38,52,57], and ZIKV could be cultured at day 7 and day 14 [38,65].

### Risk of bias in in vivo studies

Most studies did not describe in detail the methods used to avoid bias. Detailed certainty assessment of the in vivo studies is provided in S3 Table.

### Observational studies

#### ZIKV transmission between sexual partners

As of 15 April 2018, the US Centers for Disease Control and Prevention reported that, of 5,672 cases of ZIKV infection in the US, 52 were acquired through sexual transmission [72]. The European Centre for Disease Prevention and Control reported, as of 13 March 2017, 20 cases of sexual transmission out of 1,737 cases for which the route of transmission was known [73].

We included 67 reports about ZIKV sexual transmission; measurement of ZIKV infection status using RT-PCR or viral culture in samples of semen, vaginal fluid, or saliva; or both [6,20,74–138]. Twenty-four of these studies reported on 36 couples in which a primary partner with ZIKV infection, who returned from a ZIKV endemic area, is suspected to have transmitted ZIKV to a secondary partner [6,78,79,82,84,85,87,88,90,91,96,100,102,105,113,115,119–121,123,124,127,131,137] (Table 3). Thirty-four of 36 episodes of transmission were from man to woman (94%), 1 (3%) was from woman to man [96], and 1 (3%) was from man to man [85]. Penile–vaginal intercourse was reported as the most likely mode of transmission between men and women, but oral and anal intercourse were mentioned as possible transmission routes in some reports [6,78,79,84,85,88,90,91,96,100,115]. One study reported transmission of ZIKV from a vasectomised man to his female sexual partner [115]. Amongst primary infected individuals, 27/36 (75%) were symptomatic, 2/36 (6%) were asymptomatic, and symptom status was not reported for the remaining 7/36 (19%).

**Table 3:** Key characteristics of the couples (*n =* 36) for which sexual transmission of ZIKV was suspected.

Characteristic	*n* (%)	References
**Direction of transmission**		
Male–female	34 (94%)	[6,78,79,82,84,87,88,90,91,100,102,105,113,115,119–121,123,124,127,131,137]
Female–male	1 (3%)	[96]
Male–male	1 (3%)	[85]
**Symptomatic status**		
Symptomatic	27 (75%)	[6,78,79,82,84,85,88,90,96,102,113,115,121,124,127,131,137]
Asymptomatic	2 (6%)	[91,100]
Not reported	7 (19%)	[87,105,119–121,123]
**Secondary infected individual had also travelled to endemic area**		
Yes	5 (14%)	[88,91,115,124,137]
No	31 (86%)	[6,78,79,82,84,85,87,90,96,100,102,105,113,119–121,123,127,131]
**Serial interval reported**		
Yes	15 (42%)	[6,78,79,84,85,88,90,96,102,115,124,131,137]
No	21 (58%)	[82,87,91,100,105,113,119–121,123,127]
**Diagnostic certainty for primary infected individual**		
Confirmed with RT-PCR	14 (39%)	[84,85,88,90,91,96,102,115,120,121,124,127,131,137]
Confirmed with serology	4 (11%)	[6,78,79,119]
Suspected	3 (8%)	[79,100]
Not reported	15 (42%)	[82,87,105,113,121,123]
**Diagnostic certainty for secondary infected individual**		
Confirmed with RT-PCR	18 (50%)	[79,84,85,88,90,91,96,100,102,115,120,121,124,127,131,137]
Confirmed with serology	4 (11%)	[6,78,79,119]
Suspected	0 (0%)	—
Not reported	14 (39%)	[82,87,105,113,123]

RT-PCR, reverse transcription PCR.

ZIKV was detected by RT-PCR in blood, urine, saliva, or semen in 14/36 (39%) primary partners and in 18/36 (50%) secondary partners. No diagnostic method was stated for 29/72 (40%) individuals. In 5/36 (14%) couples, the secondary partner also had a history of travel to an endemic region [88,91,115,124,137].

#### Incubation period (element 2) and serial interval (element 3)

We were not able to extract information on the incubation period following sexual exposure to ZIKV, since dates of exposure of the primary partner and dates of sexual intercourse with the secondary partner were rarely reported. Thirteen reports about 15 couples reported on dates of symptom onset for both partners. The median serial interval was 12 days (interquartile range: 10–14.5 days) [6,78,79,84,85,88,90,96,102,115,124,131,137] and the maximum was 44 days [88].

#### Duration of infectiousness (element 4)

Duration of infectiousness was not measured directly in any included study. Observational studies measured the duration of detection of ZIKV in bodily fluids in case reports, case series, and prospective cohort studies. S2 Text provides additional information.

#### Case reports and case series

**Fig 3:**
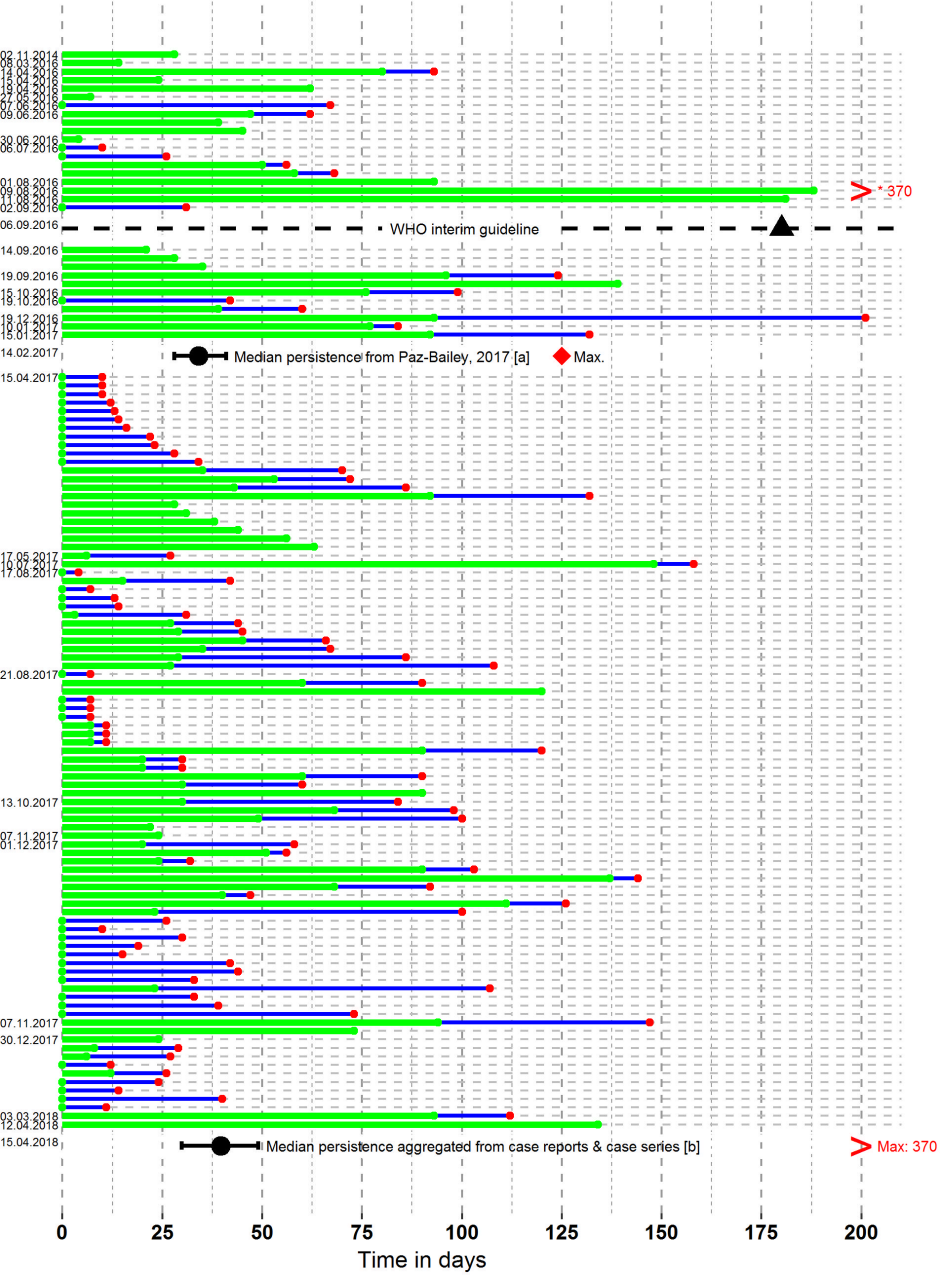
ZIKV detection in semen by RT-PCR. The *x*-axis indicates time in days from symptom onset. The labels on the *y*-axis represent the date of publication of the studies, in chronological order, with the last date indicating the date of this analysis. Green lines represent the duration of RT-PCR positivity in individuals from case reports and case series (*n =* 119), extending to the last positive RT-PCR measurement. Green dots at day 0 represent an assumption of RT-PCR positivity for patients with no sample taken at symptom onset. Blue lines represent the interval between the last positive measurement and the first subsequent negative measure (red dot). The black dotted line represents the publication of the WHO interim guidelines [2] and the suggested duration of protected sexual intercourse advised in the guidelines (6 months, black triangle). The black dots and whisker bars represent median aggregated values and 95% confidence intervals for [a] a prospective cohort (*n =* 55 men) [20] and [b] the aggregation of all available case reports and case series. Maximum values in these datasets are shown with a red diamond or a red greater than symbol (for values outside the range of the graph). Lines for which the date is not provided are from the same date as the line above. RT-PCR, reverse transcription PCR; ZIKV, Zika virus.

We included 48 publications describing 180 individuals who underwent diagnostic testing by RT-PCR or viral culture on semen, vaginal fluid, or saliva at 1 or more time points [75,76,80,81,83–86,88–95,97–100,102–104,107–117,122,124–126,128–138]. In semen (data available from 37 case reports and case series from 119 individuals; Fig 3; S2 Text), the median duration of RT-PCR positivity was 39.6 days (95% CI: 29.9–49.0 days) and the maximum was 370 days [134]. The median duration based on viral culture was 9.5 days (95% CI: 1.2–20.3 days) (data from 22 men in 11 reports), with a maximum of 69 days [115]. The median duration of ZIKV positivity in any fluid from the female genital tract was 13.9 days (95% CI: 7.2–19.6 days) based on RT-PCR (data from 15 women in 7 reports), with a maximum of 37 days [133]. The median duration of ZIKV positivity in saliva was 7.3 days (95% CI: 4.2–10.8 days) based on RT-PCR (data from 76 individuals in 23 reports), with a maximum of 91 days [99]. There were too few data for analysis of viral culture specimens in female genital tract fluids and saliva.

#### Prospective cohort studies

One cohort study enrolled 150 women and men with symptomatic ZIKV infection in Puerto Rico. ZIKV was detected by RT-PCR in 31/55 men, with a median duration of persistence of 34 days (95% CI: 28–41 days). ZIKV RNA was only detected in saliva or vaginal fluids in a few participants [20]. A second cohort study, amongst people returning from ZIKV endemic areas or infected in the US, detected ZIKA RNA in the semen of 60/184 symptomatic men [74]. The mean time to ZIKV RNA clearance was 54 days (95% CI: 53–55 days). The median duration was not reported, but plotted at approximately 35 days. Only 3 out of 19 of the semen samples provided within 30 days after symptom onset could be cultured; none of the 59 samples provided after 30 days could be cultured.

#### Risk of bias in observational studies

Studies varied widely in risk of bias and completeness of reporting (S3 Table). Many studies reporting on transmission events did not use reliable diagnostic methods in both partners, potentially leading to misclassification bias. The median duration of ZIKV persistence was higher in case reports and case series than in the prospective cohort study.

### Mathematical modelling studies

#### Reproduction number (element 5), transmission probability (element 6), and transmission rate (element 7)

We included 2 mathematical modelling studies, both of which used a deterministic structure [139,140]. Gao et al. used surveillance data from Brazil, Colombia, and El Salvador [139]; Towers et al. used data from Colombia [140]. Both studies derived the reproduction number for ZIKV sexual transmission: 0.136 (95% CI: 0.009–0.521) [139] and “likely below 1” [140]. The 2 studies calculated the proportion of ZIKV infections resulting from sexual transmission as 3.04% (95% CI: 0.12%–45.73%) [139] and 23% (95% CI: 1%–47%) [140]. Neither study provides new information about the transmission probability per sex act or the transmission rate for sexual transmission of ZIKV.

#### Risk of bias of mathematical modelling studies

For both modelling studies, the data used to populate the model were not suitable to derive the outcome. The surveillance data on which these studies based their results did not distinguish between vector-transmitted ZIKV and sexually transmitted ZIKV. The results of these studies did not provide information about the size of the risk of sexual transmission. External validation for both models is lacking. Detailed certainty assessment is shown in S3 Table.

#### Sexual transmission framework elements

Table 4 summarises findings for the outcomes of the sexual transmission framework and the GRADE assessment of the certainty of the evidence. S3 Table provides the GRADE evidence profile.

**Table 4:** Summary of the evidence on sexual transmission of Zika virus as assessed using the sexual transmission framework.

Element	Value	Sample size	References	GRADE
**1. Susceptibility**	Summary: based on animal models, rectal and vaginal mucosae are susceptible to infection. The testes form a reservoir for virus. Male–female transmission is more common than female–male transmission.	—	[32, 34–36, 40, 43, 47, 49, 51, 58, 59, 63]	NA
**2. Incubation period following sexual transmission**	Could not be calculated	—		NA
**3. Serial interval**	Median: 12 days (interquartile range: 10–14.5)	15 couples	[6, 78, 79, 84, 85, 88, 90, 96, 102, 115, 124, 131, 137]	Very low^1^
**4. Duration of infectiousness**				
Male genital tract RT-PCR (cohorts)	Median: 34 days (95% CI: 28–41)	*n =* 55	[20]	Low^2^
	Median: 35 days, mean: 54 days (95% CI: 53–55)	*n =* 184	[74]	Low^2^
Male genital tract RT-PCR (case reports and case series)	Median: 39.6 days (95% CI: 29.9–49.0)	*n =* 119	[76,80,84–86,88–95,97,98,100,102,103,107,109,112,113,115–117,124,126,128–137]	Very low^2^^,^^3^
Male genital tract viral culture	Median: 9.5 days (95% CI: 1.2–20.3)	*n =* 22	[80, 84, 86, 92, 99, 103, 107, 112, 115, 126, 130]	Very low^1^^,^^2^^,^^3^
Female genital tract RT-PCR	Median: 13.9 days (95% CI: 7.2–19.6)	*n =* 15	[95, 104, 108, 110, 111, 114, 133]	Very low^1^^,^^2^^,^^3^
Saliva RT-PCR	Median: 6.8 days (95% CI: 4.3–9.6)	*n =* 76	[75, 81, 83–85, 89, 90, 92–94, 98, 99, 104, 110, 112, 122, 125, 126, 131, 133, 134, 138]	Very low^1^^,^^2^^,^^3^
**5. Reproduction number due to sexual transmission**	<1	—	[139, 140]	Very low^4^
**6. Probability of transmission per sex act**	Could not be calculated	—		NA
**7. Transmission rate**	(Assumed)	—		NA
Proportion of cases due to sexual transmission	3.0% (95% CI: 0.1%–45.7%); 23% (95% CI: 1%–47%)	—	[139, 140]	Very low^4^

Estimates of the outcomes and publications that provide evidence for these different elements of the sexual transmission framework are listed by outcome. Additionally, the certainty assessment using Grading of Recommendations Assessment, Development and Evaluation (GRADE) methodology is provided.

^1^Small sample size or small number of studies.

^2^Indirect measure of duration of infectiousness.

^3^Risk of selection bias or selective reporting.

^4^Serious indirectness and imprecision.

NA, not applicable; RT-PCR, reverse transcription PCR.

### Other flaviviruses

We included 18 studies reporting on the sexual transmission potential of other arthropod-borne flaviviruses [14,141–157]. Ten of the 18 studies (56%) were in vitro experiments or observations in animals, and 8 studies (44%) were case reports or case series. JEV was demonstrated to be transmissible from male to female pigs via semen [141–144]. Persistence of virus was demonstrated for at least 17 days in boars [141]. JEV can be cultured from the seminal fluids of pigs [145]. In humans, we found 1 case report of male-to-female sexual transmission of WNV, although the secondary partner also lived in a mosquito endemic area [146]. WNV was found postmortem in the prostate and testis of a 43-year-old man on immunosuppressive therapy following a kidney transplant [147]. Intravaginal inoculation of WNV in mice led to local acute inflammation followed by systemic illness in a proportion of the animals [148]. The testes of 6 Japanese macaques (*M*. *fuscata*) showed low DENV neutralising antibody titres [149]. Experimentally DENV-infected pigtail macaques (*M*. *nemestrina*) showed dissemination of virus in the prostate gland and seminal vesicles [150]. DENV RNA could be detected in experimentally infected mice 3 days after infection [151]. In humans, 4 case reports describe the presence of DENV in saliva, diagnosed by either RT-PCR or viral culture, for up to 7 days [152–155]. DENV RNA was demonstrated in the vaginal secretion of 1 patient up to 18 days after onset of symptoms [156]. Female mice that were mated to male mice infected with tick-borne encephalitis virus had worse reproductive outcomes than ones mated to a group of non-infected males; in 1 female mouse, the virus was detected [157]. YFV was demonstrated in the urine and semen of a patient by RT-PCR 21 days after onset of symptoms [14].

## Discussion

This systematic review summarises published data related to sexual transmission of ZIKV and other arthropod-borne flaviviruses published on or before 15 April 2018. In animals, vaginal and rectal mucosae are susceptible to ZIKV, with the testis as a preferred site of replication. Male-to-female transmission was more frequent than female-to-male transmission in animal models and in humans. In humans, we estimated the serial interval for sexually transmitted infection to be 12 (interquartile range: 10–14.5) days. ZIKV was detectable in semen for a median of 34 (95% CI: 28–41) days by RT-PCR and 9.5 (95% CI: 1.2–20.3) days by viral culture. In mathematical modelling studies, the reproduction number for sexual transmission of ZIKV was below 1. The overall certainty of the evidence was low. We found no evidence that other arthropod-borne flaviviruses can be sexually transmitted.

### What the study adds to existing research

The ZIKV sexual transmission framework allowed us to synthesise evidence from both animal and human studies in a structured way, taking into account the risks of bias in the included studies. Susceptibility of tissues to ZIKV could only be assessed in animal models. There were consistent findings in animal models that help to explain the overrepresentation of reported cases of human male-to-female transmission, even though mice are not a natural host for ZIKV and in vivo studies often use immunocompromised animals [8]. First, vaginal mucosae are more susceptible than urethral mucosae to infection [36,40,47,49,51,59]. Second, high levels of ZIKV replication in the testes in mice and sustained detection of viral RNA and of virus in tissue culture in mice and NHP models are consistent with the longer duration of detection in men than women. Rectal mucosa is also susceptible to ZIKV, so, although only observed once [85], unprotected anal intercourse is also a likely route of ZIKV transmission. The risk of bias of the included in vivo studies, as assessed with the SYRCLE tool, was high. Most of these studies explored the suitability of animal models or investigated pathophysiological pathways, and potential sources of bias were rarely reported.

Our analysis shows that, when assessed from case reports and case series, the duration of detection of ZIKV in semen by RT-PCR is overestimated; all reports are of people with ZIKV detected, and a small number of outliers influence the estimate. A prospective cohort study that consecutively enrolled people with symptomatic ZIKV infection estimated a shorter duration of persistence [22]. Case reports and case series are early sources of information about a new disease, but, by the nature of these studies, researchers report novel and unusual findings. Parameters and effect sizes estimated from aggregating data from these sources are likely to be overestimates, reflecting the so-called random high: extreme values in a distribution that are observed by chance and are more likely to be reported because they are noteworthy [158]. As evidence has accumulated in well-designed prospective studies, the estimated duration of persistence of ZIKV RNA has decreased. Notably, the prospective cohort study in Puerto Rico found ZIKV in semen in only half of men with symptomatic infection and vaginal fluid in only 1 of 50 women. Similarly, ZIKV was found in semen in only 60/183 (33%) ZIKV-infected men in the US [74]. Persistence of viral RNA in body fluids is often used as a proxy for the duration of ZIKV infectiousness, although it remains unclear whether the presence of viral RNA corresponds with infectious virus. ZIKV RNA positivity persists for longer than detection of ZIKV in viral culture in both mice [47] and human semen samples. However, viral cultures might underestimate the duration of infectiousness if low pH or other specimen-dependent factors produce false negative results [83,159]. The estimated serial interval was based on observations from only 15 couples, but was consistent with that of several respiratory infectious diseases [160]. The serial interval for sexual transmission was towards the lower end of estimates for mosquito-borne transmission (10–23 days) [161].

Some elements of the infection process, such as the incubation period, transmissibility of ZIKV per sex act, and transmission rate could not be observed. In the mathematical models published so far [139,140,162], the estimates were based on assumptions about the transmissibility of mosquito-borne infection. Estimates from our review might provide more reliable data for use in future modelling studies. The potential for sustained sexual transmission of ZIKV appears low, based on the reproduction number estimated in the mathematical modelling studies. The estimated reproduction number was higher for mosquito-borne transmission, 1.96 (95% CI: 0.45–6.23) than for sexual transmission [139], although this number is highly dependent on the geographical location [163].

This review did not find evidence supporting sexual transmission of other arthropod-borne flaviviruses. The continual updating of the literature search identified a finding of YFV in urine and semen [14]. However, it remains to be clarified for many viruses if detection in semen means that there is a risk of sexual transmission [13].

### Strengths and weaknesses of the study

The strengths of our living systematic review are the high coverage of the body of published literature, the structured overview, and the reanalysis of individual patient data on persistence of ZIKV. The automation of search and deduplication processes makes it feasible to keep the review updated as new information becomes available. Updated analyses of the data from case reports show regression to the mean of the median estimate of the duration of RNA detection in semen (https://zika.ispm.unibe.ch/stf/). Future updates of this review will also allow for incorporation of techniques to synthesise mathematical modelling studies, such as multi-model ensembles. This study also has limitations. Screening and data extraction were not done by 2 independent reviewers because of time constraints, but we believe that we reduced errors by having a second reviewer check the decisions and data extracted. The statistical methods used to estimate the duration of persistence of ZIKV in bodily fluids assume that all samples are positive for ZIKV at time 0 [20], which might not be the case. Additionally, the sexual transmission framework might not include all factors that are required to investigate the risks of sexual transmission of ZIKV. The certainty of this body of evidence was assessed as being low or very low because of bias in the observational study designs and the indirectness of evidence from animal studies. The certainty of the evidence base could increase if the design and reporting of both animal and human studies improve and if their findings are consistent with, and increase the precision of, the evidence presented here.

### Implications and next steps for researchers, clinicians, and policymakers

The risk of sexual transmission of ZIKV is particularly relevant for women who are pregnant or planning a pregnancy and for people with high levels of sexual partner change such as some groups of men who have sex with men and women at high risk. An expert group has used the ZIKV sexual transmission framework to stimulate discussion about research priorities [17]. One important limitation to the generalisability of findings from our review is that the data that we analysed about sexual transmission of ZIKV in humans relied largely on information from travellers returning from endemic areas with symptomatic ZIKV infection and their sexual partners. This group probably differs from people in endemic regions in ways that could affect sexual transmission of ZIKV, such as previous exposure to other flaviviruses [164]. Additional studies in ZIKV endemic settings could enrol travellers who work in areas with mosquito-borne ZIKV transmission and who return to families living nearby but in areas, e.g., at high altitude, where the vector does not survive [17]. There are unanswered questions about the potential for asymptomatic ZIKV infection following sexual transmission, since ZIKV transmitted by mosquitoes often causes asymptomatic infection [165–167], about clinical differences between ZIKV infections acquired through sexual and mosquito-borne routes, and about the long-term consequences of ZIKV in the genital tract, such as its effects on the testes and on male infertility. Research about the potential for sexual transmission of other flaviviruses is needed, although these viruses often display different symptomatology or affinity for different species.

Clinicians and policymakers need information that helps to advise both opposite sex and same sex couples on how to reduce the risk of sexual transmission of ZIKV. The relationship between detectable RNA in semen and infectiousness therefore needs to be further investigated in both laboratory and epidemiological studies. Current guidelines for travellers returning from endemic areas advise 6 months of protected intercourse [1,2]. As more information becomes available, a revision of the duration of protection might be indicated.

## Conclusions

This living systematic review gives an up-to-date synthesis of information about the sexual transmission of ZIKV with a structured framework. Planned regular updates will allow timely updating of relevant data from a rapidly expanding evidence base. We did not quantify the absolute risk of sexual transmission of ZIKV, but it appears small based on information about the proportion of people with symptomatic ZIKV who have ZIKV detected in genital secretions and the short median duration of detection of ZIKV in semen and vaginal fluid. Taking into account all available data about the duration of detection of ZIKV in culture and from the serial interval, our findings suggest that the infectious period for sexual transmission of ZIKV is shorter than estimates from the earliest post-outbreak studies, which were based on RT-PCR alone.

## Supporting information

S1 PRISMA Checklist (DOCX)

S1 Table Detailed description of case reports and case series describing sexual transmission of ZIKV. (XLSX)

S2 Table Detailed description of in vivo and in vitro studies. (XLSX)

S3 Table Certainty assessment of outcomes and studies. (XLSX)

S1 Text Living systematic review protocol. (DOCX)

S2 Text Methodology of survival analysis. (DOCX)

## Abbreviations

DENV: dengue virus
GRADE: Grading of Recommendations Assessment, Development and Evaluation
JEV: Japanese encephalitis virus
NHP: nonhuman primate
RT-PCR: reverse transcription polymerase chain reaction
SYRCLE: SYstematic Review Centre for Laboratory animal Experimentation
WNV: West Nile virus
YFV: yellow fever virus
ZIKV: Zika virus
