# Supplementary material for: Sexual transmission of Zika virus and other flaviviruses: A living systematic review
Source: PLoS Med. 2018 Jul 24;15(7):e1002611. doi: 10.1371/journal.pmed.1002611 (PMC6057622; doi:10.1371/journal.pmed.1002611)
Supplement: S1 Text — (DOCX) [file pmed.1002611.s005.docx]

# S2 Text. Living systematic review protocol

A living systematic review is a review that is updated continually [1]. In order to keep up with the emerging evidence, we plan to update the review according to the description below. The protocol below is based on [2]. We describe the automation and implementation of the living systematic review elsewhere [3]

## Search strategy

A list of arthropod borne flaviviruses was compiled from [2, 4-6] and pilot searches were performed on Pubmed using the virus name and terms related to sexual transmission. Viruses that yielded results were maintained in the search strategy.

**OVID:**

1. sperm/ or vagina/ or testis/ or saliva/
2. (Sexual* or semen or sperm or seminal or saliva or vaginal* or testis or genital).ti,ab
3. 1 or 2
4. flavivirus/ OR dengue virus/ OR encephalitis viruses, japanese/ OR yellow fever virus/ OR zika virus/ OR flavivirus infections/ OR dengue/ OR encephalitis, japanese/ OR "St. Louis encephalitis"/ OR west nile fever/ OR yellow fever/ OR zika virus infection/
5. (Dengue OR Flavivirus OR Jiangsu virus OR Japanese encephalitis OR Murray Valley encephalitis OR St Louis encephalitis OR Usutu virus OR Xishuangbanna virus OR Yaounde virus OR West Nile OR yellow fever OR zika OR zika virus).ti,ab,kw.
6. 4 or 5
7. 3 and 6

**Pubmed:**

1. Semen[mesh] or vagina[mesh] or testis[mesh] or saliva[mesh]
2. (sexual*[tiab] or semen[tiab] or sperm[tiab] or seminal[tiab] or saliva[tiab] or vaginal[tiab] or testis[tiab] or genital tract[tiab])
3. 1 or 2
4. Flavivirus[mesh] OR dengue virus[mesh] OR encephalitis viruses, japanese[mesh] OR yellow fever virus[mesh] OR zika virus[mesh] OR flavivirus infections[mesh] OR dengue[mesh] OR encephalitis, japanese[mesh] OR encephalitis, st. louis[mesh] OR west nile fever[mesh] OR zika virus infection[mesh]
5. Dengue[tiab] OR Flavivirus[tiab] OR Jiangsu virus[tiab] OR Japanese encephalitis[tiab] OR Murray Valley encephalitis[tiab] OR St Louis encephalitis[tiab] OR Usutu virus[tiab] OR Xishuangbanna virus[tiab] OR Yaounde virus[tiab] OR West Nile[tiab] OR yellow fever[tiab] OR zika virus[tiab] OR zika[tiab]
6. 4 or 5
7. 3 and 6

**Embase:**

1. 'sperm'/exp OR 'vagina'/exp OR 'testis'/exp OR 'saliva'/exp
2. sexual*:ti,ab OR semen:ti,ab OR sperm:ti,ab OR seminal:ti,ab OR saliva:ti,ab OR vaginal*:ti,ab OR testis:ti,ab
3. #1 or #2
4. 'Flavivirus'/exp
5. dengue:ti,ab OR flavivirus:ti,ab OR 'jiangsu virus':ti,ab OR 'japanese encephalitis':ti,ab OR 'murray valley encephalitis':ti,ab OR 'st louis encephalitis':ti,ab OR 'usutu virus':ti,ab OR 'xishuangbanna virus':ti,ab OR 'yaounde virus':ti,ab OR 'west nile':ti,ab OR 'yellow fever':ti,ab OR zika:ti,ab OR 'zika virus':ti,ab
6. #4 or #5
7. #3 and #6

**LILACS:**

(tw:(sexual) or tw:(semen) or tw:(sperm) or tw:(seminal) or tw:(saliva) or tw:(vaginal) or tw:(testis) or tw:(genital tract)) AND (tw:(Dengue) OR tw:(Flavivirus) OR tw:(Jiangsu virus) OR tw:(Japanese encephalitis) OR tw:(Murray Valley encephalitis) OR tw:(St Louis encephalitis) OR tw:(Usutu virus) OR tw:(Xishuangbanna virus) OR tw:(Yaounde virus) OR tw:(West Nile) OR tw:(yellow fever) OR tw:(zika virus) OR tw:(zika))

## Databases and other information sources

**Table 1. Databases and other information sources used in the systematic review.**

| **Database** | **Url** |
| --- | --- |
| Embase | <https://www.embase.com/> |
| Pubmed | <https://www.ncbi.nlm.nih.gov/pubmed/> |
| LILACS | [http://bases.bireme.br/cgi-bin/wxislind.exe/iah/online/ ?IsisScript=iah/iah.xis&base=LILACS&lang=i&form=F](http://bases.bireme.br/cgi-bin/wxislind.exe/iah/online/%20?IsisScript=iah/iah.xis&base=LILACS&lang=i&form=F) |
| BioRxiv | <https://biorxiv.org/> |
| Arxiv | <https://arxiv.org/> |
| PeerJ | <https://peerj.com/> |
| WHO | <http://www.who.int/csr/disease/zika/en/> |
| PAHO | <http://www.paho.org/hq/index.php?option=com_content>  [&view=article&id=11585&Itemid=41688&lang=en](http://www.paho.org/hq/index.php?option=com_content&view=article&id=10898&Itemid=41443&lang=en) |
| ECDC | <https://ecdc.europa.eu/en/zika-virus-infection> |
| CDC | <https://www.cdc.gov/zika/index.html> |

## Searching and screening frequency

We will perform automated daily searches for Pubmed, EMBASE and LILACS. We will perform monthly searches for the remaining information sources (table 1). We will screen any new citations retrieved by the searches immediately. When items are retained, we will extract data and perform bias assessment.

## Data synthesis and dissemination

We will re-analyse and update the tables and figures specified in Table 2 every three months. We will publish these updates on a designated website (<https://zika.ispm.unibe.ch/stf/>), and summarize the findings in the comments section of the publication.

**Table 2. Elements that will be updated using the living systematic review protocol.**

| **Table/figure** | **Description** |
| --- | --- |
| Figure 2 | Flow diagram of reviewed studies. |
| Table 2 | Overview of study designs of included studies. |
| Table 3 | Key characteristics of the couples for which sexual transmission of ZIKV was suspected. |
| Figure 3 | Semen RT-PCR results from individual patients and aggregated data. |
| Table 4 | Summary of the evidence on sexual transmission of Zika virus as assessed using the sexual transmission framework. |
| S3 Text - Figure 1 | Weibull survival curves of the duration of ZIKV positivity. |
| S3 Text - Table 1 | Duration of ZIKV positivity - available data per outcome. |
| S4 Table | Detailed description of couples. |
| S5 Table | Detailed description of *in vivo*/*in vitro* studies |
| S6 Table | Quality assessment of included studies. |

## Duration of maintenance of the living systematic review

We will keep the living systematic review up to date for as long as new relevant data comes out and to a maximum of the duration of the project funding (Swiss National Science Foundation [Grant number: 320030_170069], end date: 31/10/2021 - <http://p3.snf.ch/project-176233>)

## References

1. Elliott JH, Turner T, Clavisi O, Thomas J, Higgins JP, Mavergames C, et al. Living systematic reviews: an emerging opportunity to narrow the evidence-practice gap. PLoS Med. 2014;11(2):e1001603. doi: 10.1371/journal.pmed.1001603. PubMed PMID: 24558353; PubMed Central PMCID: PMC3928029.

2. Synnot A, Turner T, Elliott J, Living Systematic Review Network. Cochrane Living Systematic Reviews Interim guidance for pilots (Version 0.3, 21 April 2017) 2017 [Access Date:16/04/2018]. Available from: <http://community.cochrane.org/sites/default/files/uploads/inline-files/Transform/LSR%20Interim%20guidance_v0.3_20170703.pdf>.

3. Counotte MJ, Egli-Gany D, Riesen M, Abraha M, Porgo TV, Wang J, et al. Zika virus infection as a cause of congenital brain abnormalities and Guillain-Barré syndrome: From systematic review to living systematic review. F1000Research. 2018;7.

4. Duggal NK, McDonald EM, Ritter JM, Brault AC. Sexual transmission of Zika virus enhances in utero transmission in a mouse model. Sci Rep. 2018;8(1):4510. Epub 2018/03/16. doi: 10.1038/s41598-018-22840-6. PubMed PMID: 29540804; PubMed Central PMCID: Pmc5852059.

5. Chambers TJ, Hahn CS, Galler R, Rice CM. Flavivirus genome organization, expression, and replication. Annual review of microbiology. 1990;44:649-88. Epub 1990/01/01. doi: 10.1146/annurev.mi.44.100190.003245. PubMed PMID: 2174669.

6. Kuno G, Chang GJ, Tsuchiya KR, Karabatsos N, Cropp CB. Phylogeny of the genus Flavivirus. J Virol. 1998;72(1):73-83. Epub 1998/01/07. PubMed PMID: 9420202; PubMed Central PMCID: PMC109351.
