# Supplementary material for: Sexual transmission of Zika virus and other flaviviruses: A living systematic review
Source: PLoS Med. 2018 Jul 24;15(7):e1002611. doi: 10.1371/journal.pmed.1002611 (PMC6057622; doi:10.1371/journal.pmed.1002611)
Supplement: S2 Text — (DOCX) [file pmed.1002611.s006.docx]

# S3 Text. Methodology of survival analysis.

## Inclusion of studies

To estimate the duration of infectiousness, we used the duration of detection of Zika virus (ZIKV) in different bodily fluids as a proxy. We included studies that conducted diagnostic testing of ZIKV using reverse transcription polymerase chain reaction (RT-PCR) and/or viral culture on saliva, fluids from the female genital tract or semen. Studies were included when testing was performed at one or more well defined time points. Time points were defined as days post onset of symptoms, limiting inclusion to symptomatic patients.

## Interval censored survival analysis

Due to the scarcity of data, we grouped measurements in cervical and vaginal fluid (hereafter referred to as 'female genital tract'). To aggregate the duration of positivity we applied survival analysis. Since the exact duration of ZIKV positivity diagnosed by RT-PCR or viral culture was unknown, we applied interval censored survival analysis. The survival event was defined as becoming ZIKV negative. All subjects were assumed to be positive at day 0, similar to the methodology used in [1]. Patients with a ZIKV positive result followed by a ZIKV negative result at a later time point, were assumed to become negative at the interval between both results; patients with only ZIKV negative results were assumed to become negative between day 0 and the first negative test; patients with only ZIKV positive measurements were considered right censored. We applied a Weibull proportional hazards model for interval censored survival data using the 'straweib' package [2, 3] in R [4], allowing the fitting of a Weibull regression model to the extracted data using maximum likelihood estimation and its associated pointwise 95% confidence intervals. Fig 1 provides Weibull survival curves of the duration of ZIKV positivity for the different bodily fluids. Fig 2 provides the interval censored survival Kaplan-Meier curve (blue) and the fitted Weibull curve (black) and the 95% confidence intervals (dashed). Table 1 provides additional information on the data used.

**
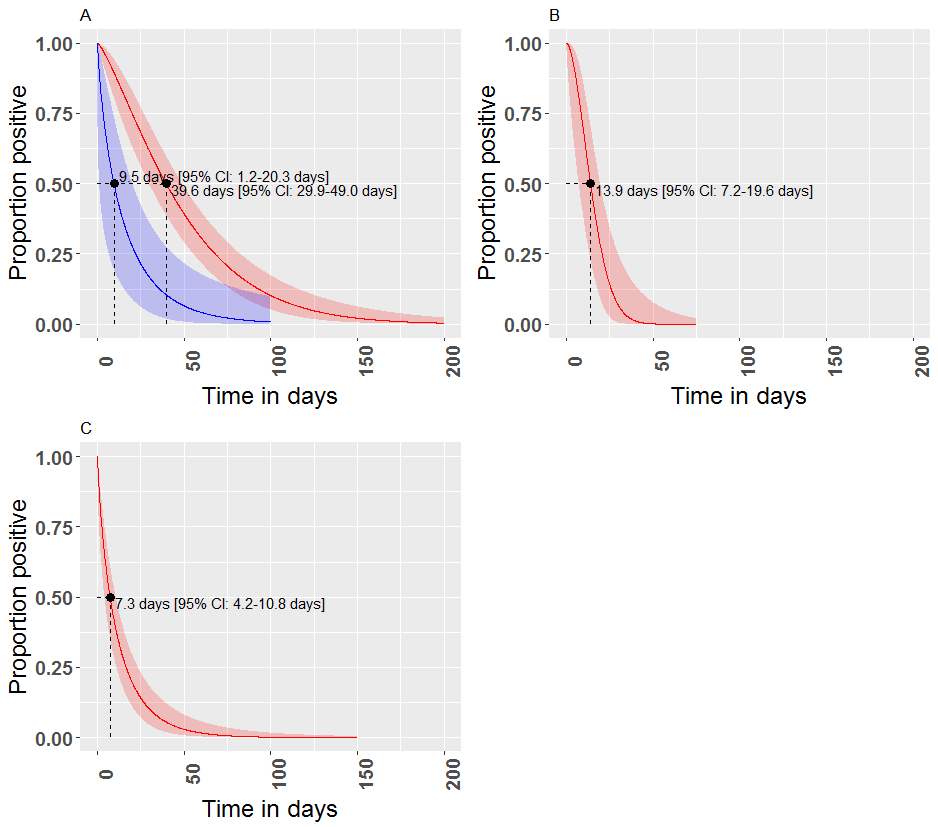
**

**Fig 1. Weibull survival curves of the duration of ZIKV positivity.** Semen (panel A), female genital fluids (panel B), and saliva (panel C) diagnosed with RT-PCR (red curve) and viral culture (blue curve).

| A | B |
| --- | --- |
| 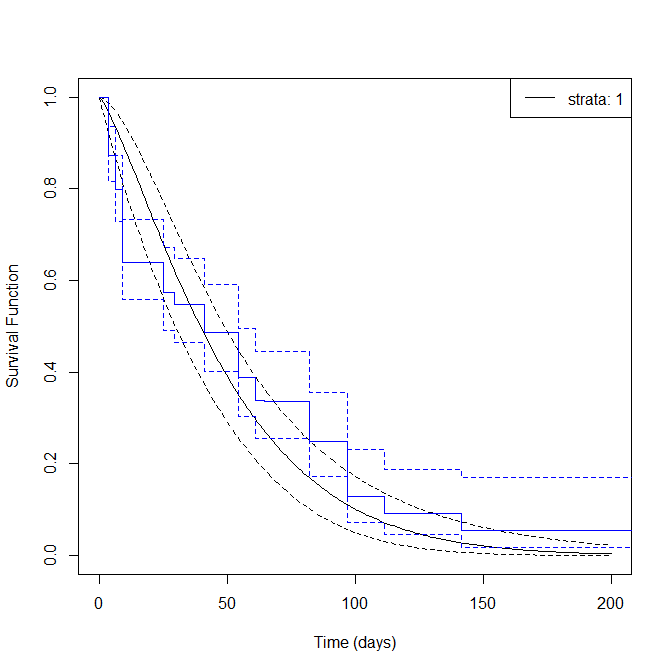 | 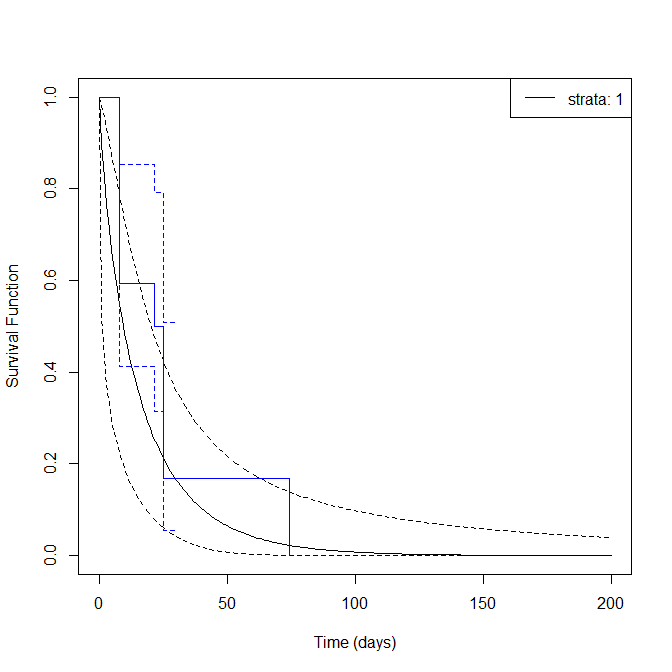 |
| C | D |
| 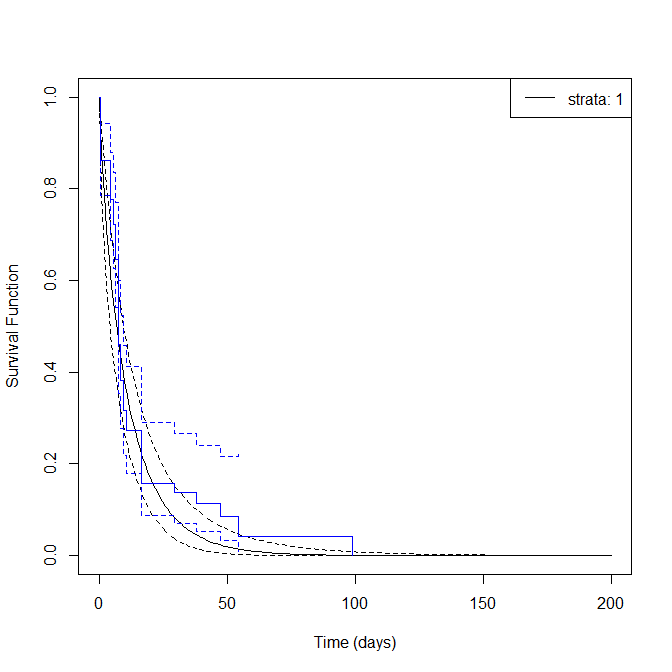 | 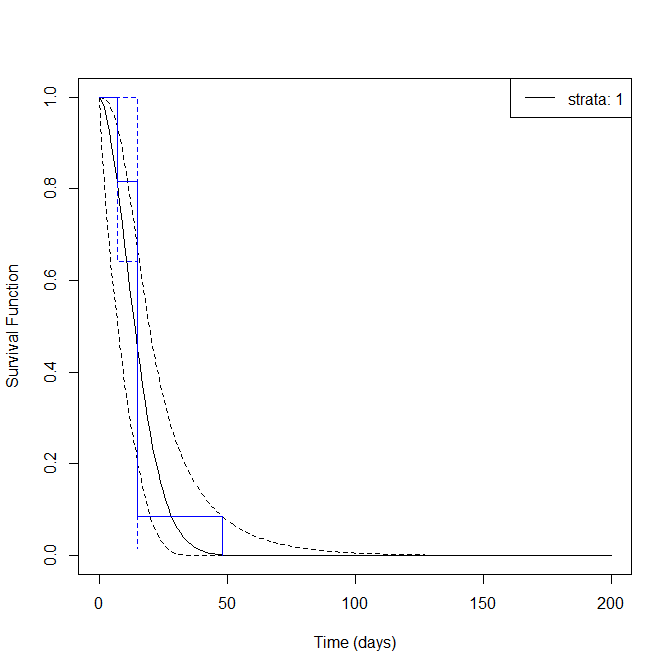 |

**Fig 2. Interval censored Kaplan-Meier survival curves (blue) and Weibull fitted curves (black).** Dashed lines represent 95% confidence intervals. Panel A. RT-PCR semen, panel B. viral culture semen, panel C. RT-PCR saliva, and panel D. RT-PCR for female genital tract.

**Table 1. Duration of ZIKV positivity - available data per outcome per 15/04/2018.**

| **Fluid** | **Method** | **# Patients** | **# References** |
| --- | --- | --- | --- |
| Semen | RT-PCR | 114 | 35 |
|  | viral culture | 22 | 11 |
| Saliva | RT-PCR | 67 | 20 |
|  | viral culture | 4 | 4 |
| Female genital tract | RT-PCR | 15 | 7 |
|  | viral culture | 0 | 0 |

## References

1. Paz-Bailey G, Rosenberg ES, Doyle K, Munoz-Jordan J, Santiago GA, Klein L, et al. Persistence of Zika Virus in Body Fluids - Preliminary Report. N Engl J Med. 2017. doi: 10.1056/NEJMoa1613108. PubMed PMID: 28195756.

2. Gomez G, Calle ML, Oller R, Langohr K. Tutorial on methods for interval-censored data and their implementation in R. Stat Model. 2009;9(4):259-97. doi: 10.1177/1471082x0900900402.

3. Gu X, Shapiro D, Hughes MD, Balasubramanian R. Stratified Weibull Regression Model for Interval-Censored Data. R J. 2014;6(1):31-40. PubMed PMID: 26835159; PubMed Central PMCID: PMC4729374.

4. R. Core Team. R: A Language and Environment for Statistical Computing. Vienna, Austria: R Foundation for Statistical Computing; 2017 2017.
